# Identification of Nanoplastics by Probing the Viscous Nanoenvironment

**DOI:** 10.1002/smsc.202500430

**Published:** 2025-11-10

**Authors:** Liang Li, Wenjie Yang, Yonggen Hong, Qiyuan He, Xuanyi Lu, Hong Wang, Peng Tao, Chao Shu, Mingqing Chen, Guochen Bao, Lijun Jiang

**Affiliations:** ^1^ Key Laboratory of Pesticide and Chemical Biology of Ministry of Education Hubei Key Laboratory of Genetic Regulation & Integrative Biology School of Life Sciences Central China Normal University Wuhan 430079 China; ^2^ Department of Applied Biology and Chemical Technology The Hong Kong Polytechnic University Hong Kong SAR China; ^3^ State Key Laboratory of Green Pesticide, College of Chemistry Central China Normal University Wuhan 430079 China; ^4^ School of Mathematical and Physical Sciences, Faculty of Science University of Technology Sydney Sydney New South Wales 2007 Australia

**Keywords:** fluorescence, imaging, labeling, molecular probes, nanoplastics

## Abstract

With the growing prevalence of global microplastic and nanoplastic pollution, the accumulation of nanoplastics in the human body has increased, heightening the risk of noncommunicable diseases including cancer, cardiovascular disease, and amyotrophic lateral sclerosis. However, the development of fluorescent probes for detecting nanoplastics remains challenging due to the lack of reactive sites on nanoplastics for conventional design of responsive probes. In this work, a novel strategy for the sensitive detection of nanoplastics by probing the viscous nanoenvironment surrounding them is presented. This study synthesizes a cationic fluorescent probe, Purification by silica gel column chromatography (CH_2_Cl_2_/MeOH) provided (E)‐2‐(2‐(4‐(dimethylamino)nanphthalen‐1‐yl)vinyl)‐1,3,3‐trimethyl‐3H‐indol‐1‐ium (named HCY due to its structural similarity to hemicyanine dyes) as a tawny solid (HCY), via a simple one‐step reaction. HCY demonstrates high sensitivity to nanoplastics, achieving an 8.5‐fold fluorescence enhancement in the presence of carboxylated polystyrene nanoplastics, with a detection limit of 0.153 μg mL^−1^. Moreover, HCY exhibits excellent biocompatibility, enabling the monitoring of nanoplastics level in living cells and visualization of nanoplastics distribution in zebrafish. This work offers a new design strategy for responsive fluorescent probes and provides a promising avenue for detecting environmental pollutants.

## Graphical abstract

An approach for the sensitive detection of nanoplastics is developed by probing the viscous nanoenvironment. The probe, HCY, shows a high sensitivity to nanoplastics, with a detection limit of 0.153 μg mL^−1^. It exhibits excellent biocompatibility, allowing the monitoring of nanoplastic levels in living cells and the visualization of their distribution in zebrafish.

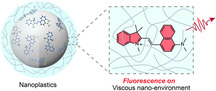

## 1 Introduction

Plastic pollution has become a major global environmental challenge, attracting widespread attention.^[^ ^1^ ^]^ Each year, over 8 million tons of plastic wastes enter the oceans worldwide.^[^ ^2^ ^]^ Microplastics and nanoplastics (collectively referred to as MNPs), generated through the physical and biological degradation of larger plastic debris, are increasingly prevalent in natural environments. Evidence suggests that contamination with MNPs is associated with various health issues, including cardiovascular diseases such as atherosclerosis,^[^ ^3^ ^]^ reproductive disorders,^[^ ^4^ ^]^ and immune and inflammatory responses.^[^ ^5^, ^6^ ^]^ Nanoplastics, in particular, pose a greater toxicity risk than larger plastic particles because their small size enables them to infiltrate biological tissues.^[^ ^7^, ^8^ ^]^ Research has shown that nanoplastic pollution can accelerate the aggregation of alpha‐synuclein, a process linked to neurodegenerative diseases such as Parkinson's disease and dementia.^[^ ^9^ ^]^ Additionally, nanoscale polystyrene (PS) has been found to induce amyotrophic lateral sclerosis‐like symptoms.^[^ ^10^ ^]^

Fluorescent probes are notable for their rapid response, high sensitivity, and ease of use. They detect analytes through various reactions or interactions,^[^ ^11^, ^12^ ^]^ which induce changes in their structures or energy transfer pathways, resulting in the alterations to fluorescent signals. Fluorescent probes are widely employed for the detection of metal cations,^[^ ^11^, ^13^, ^14^ ^]^ nonmetal ions,^[^ ^15^, ^16^ ^]^ pH levels,^[^ ^17^, ^18^ ^]^ small molecules,^[^ ^19^, ^20^, ^21^ ^]^ and biomacromolecules.^[^ ^22^, ^23^, ^24^ ^]^ However, developing fluorescent probes for nanoplastics detection remains a significant challenge, as the structure of nanoplastics typically lacks reactive sites for reactions to induce spectral change in the probes or orbital interactions necessary to influence energy transfer processes between the probes and analytes.

**Figure 1:**
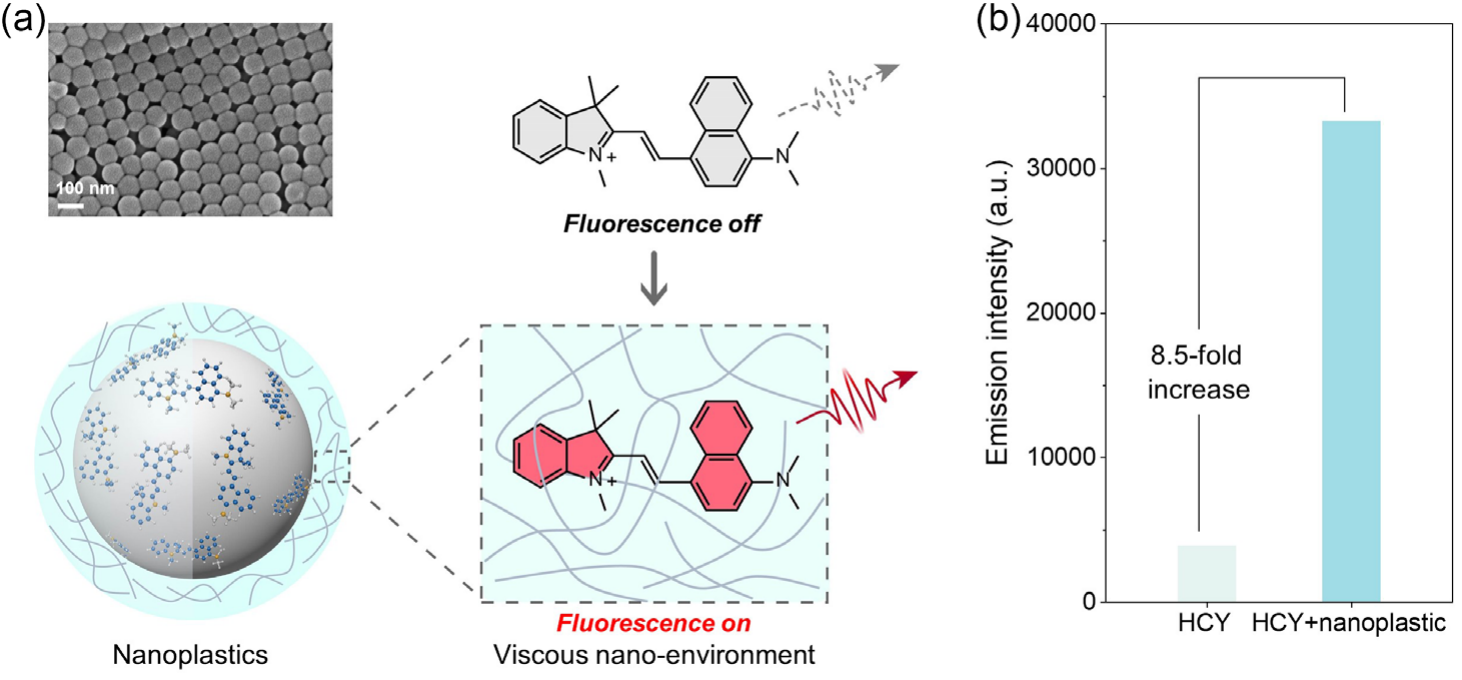
Probing the viscous nanoplastic by HCY. a) SEM image of nanoplastics and schematic illustration of the sensing mechanism of HCY to nanoplastics. b) Emission intensity of HCY at 645 nm in the absence and presence of PS‐COOH, displays the emission responsiveness to nanoplastics.

Here, we present a novel approach for the sensitive detection of nanoplastics by probing the viscous nanoenvironment surrounding them (**Figure** **1**). We synthesized a cationic fluorescent probe, HCY, through a one‐step reaction (Scheme S1, Supporting Information). Several types of model nanoplastics, including PS, polyethylene terephthalate (PET), and polypropylene (PP), were employed to study the interaction and sensing of HCY toward nanoplastics. Interactions of HCY with nanoplastics are primarily driven by restricted intramolecular motion. In addition, electrostatic adsorption contributes selectively to negatively charged nanoplastics and *π*–*π* stacking operates exclusively for those containing aromatic rings. The nanoenvironment surrounding the nanoplastics is highly viscous, which restricts the intramolecular motion of HCY and suppresses nonradiative transitions of its excited state electrons (Figure 1a). As a result, the presence of carboxylated polystyrene nanoplastics (PS‐COOH) induces an 8.5‐fold increase in the fluorescence emission intensity of HCY (Figure 1b). The probe exhibits biocompatibility, allowing for the detection and tracking of PS‐COOH nanoplastics in cells and living organisms. Our proof‐of‐concept study provides a new strategy for designing responsive fluorescent probes for nanoplastics.

## 2 Results and Discussion

### 2.1 Synthesis and Characterization

**Figure 2:**
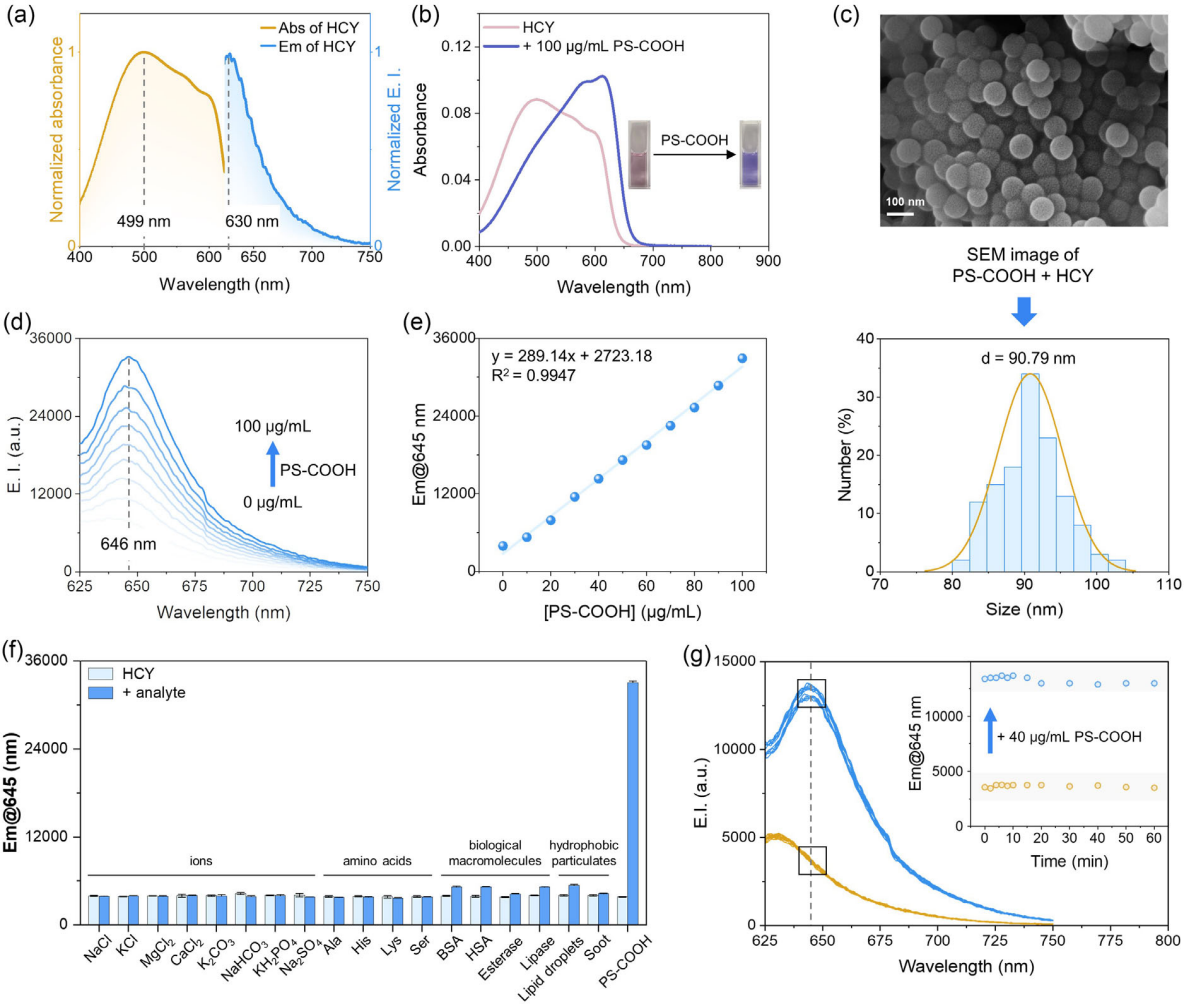
Fluorescence response of HCY to PS‐COOH nanoplastics. a) Normalized absorption and emission spectra of HCY. b) Absorption spectra of HCY with or without PS‐COOH. c) SEM image and size distribution of PS‐COOH binding with HCY. d) Emission profile of HCY with PS‐COOH ranging from 0 to 100 μg mL^−1^. e) Linearity between emission intensity of HCY at 645 nm and concentrations of PS‐COOH from 0 to 100 μg mL^−1^. f) Emission intensity of HCY alone and with different analytes (mean ± SD, *n* = 3). Concentration for all analytes is 100 μg mL^−1^. g) Emission profile of HCY alone and upon incubation with 40 μg mL^−1^ PS‐COOH as a function of time (0–60 min). *λ*_ex_  = 615 nm; [HCY] = 5 μM.

HCY was synthesized via a one‐step condensation reaction between the aldehyde group of 4‐dimethylamino‐1‐naphthaldehyde and 1,2,3,3‐tetramethyl‐3H‐indolium iodide under heating conditions. The structure of HCY was confirmed by ^1^H NMR, ^13^C NMR, and HR‐MS analyzes (Figures S1−S3, Supporting Information). HCY exhibited a broad absorption band centered at 499 nm and an emission band at 630 nm, featuring a large Stokes shift of 131 nm (**Figure** **2**a).

### 2.2 Fluorescence Response of HCY to Carboxylated Polystyrene Nanoplastics

To evaluate the sensing capacity of HCY, a representative PS nanoplastics, PS‐COOH,^[^ ^9^, ^25^, ^26^, ^27^, ^28^ ^]^ were selected and measured to check the influence on HCY emission (Figure 1b). In the presence of PS‐COOH, the absorption spectrum of HCY exhibited a significant red‐shift from 499 nm to 613 nm, accompanied by an immediate color change from pink to purple–blue, visible to the naked eye (Figure 2b). Scanning electron microscopy (SEM) images of PS‐COOH before and after interaction with HCY revealed that the binding of HCY with nanoplastics does not significantly alter their morphology and size (Figure 2c, Figure S4, Supporting Information). The fluorescence intensity of HCY increased with the rising concentration of nanoplastics, showing a satisfactory linear relationship. An 8.5‐fold fluorescence enhancement accompanied by a red‐shift from 630 nm to 646 nm was observed for this negatively charged PS‐COOH (Figure 2a,d). The limit of detection (LOD) was determined to be 0.153 μg mL^−1^, showing the well performance of HCY in detection sensitivity (Figure 2e, Table S1, Supporting Information).

To assess the selectivity of the HCY probe for nanoplastics, we evaluated the effect of amino acids (including alanine, histidine, lysine, and serine) and typical ions (including Na^+^, K^+^, Ca^2+^, Mg^2+^, Cl^−^, CO_3_^2−^, HCO_3_^−^, H_2_PO_4_^−^, SO_4_^2−^) commonly present in living organisms. Among all these analytes evaluated, HCY exhibited fluorescent response exclusively to PS nanoplastics (Figure 2f). To further evaluate whether HCY responds to biological macromolecules or hydrophobic particulates, we assessed the effect of esterase, lipase, bovine serum albumin, human serum albumin (HSA), lipid droplets, and soot. Notably, HSA is the most abundant circulating protein in blood plasma. The addition of these substances did not induce significant changes in HCY fluorescence, suggesting their presence does not interfere with the detection of nanoplastics by HCY (Figure 2f). Considering the trace surfactants are common in real samples, we investigated the effect of the nonionic surfactants Tween 20 and Tween 80 on the fluorescence response of HCY to nanoplastics. The emission of HCY, in the absence and presence of PS‐COOH, was measured in PBS, PBS containing 0.1% Tween 20, and PBS containing 0.1% Tween 80. Although the surfactants increased HCY fluorescence, the addition of PS‐COOH nanoplastics produced a further, marked fluorescence enhancement, showing that HCY is capable of detecting nanoplastics in the presence of trace surfactants (Figure S5, Supporting Information). We next examined the response time of HCY to PS‐COOH and observed an immediate fluorescence increase within 1 min (Figure 2g). The fluorescence intensity remained stable, showing no significant changes over 60 min after HCY binding to PS‐COOH (Figure 2g).

Tolerance to pH changes is critical for a fluorescent probe to be used for monitoring in environmental and physiological conditions. To assess the pH tolerance of HCY, we evaluated its fluorescence emission in the presence of PS‐COOH across a range of pH values. The fluorescence intensity of HCY remained nearly unchanged over the pH range of 4.6–9.9, demonstrating its suitability for monitoring PS‐COOH under various conditions, including physiological environments. Furthermore, the binding of HCY to PS‐COOH and the fluorescence response were unaffected by pH (Figures S6 and S7, Supporting Information).

### 2.3 Detection Mechanism of HCY

We next analyzed the detection mechanism of HCY to nanoplastics. The HCY molecule consists of an electron‐accepting indole cation connected to 4‐dimethylamino‐1‐naphthaldehyde via a rotatable vinyl bond. The electron‐donating dimethylamino group induces nonradiative transitions through intramolecular motion in solution, resulting in weak fluorescence. In the presence of nanoplastics, HCY is adsorbed on the nanoplastic surface. The viscous nanoenvironment surrounding nanoplastics suppresses the intramolecular motion and nonradiative transitions, leading to a significant fluorescence enhancement.

**Figure 3:**
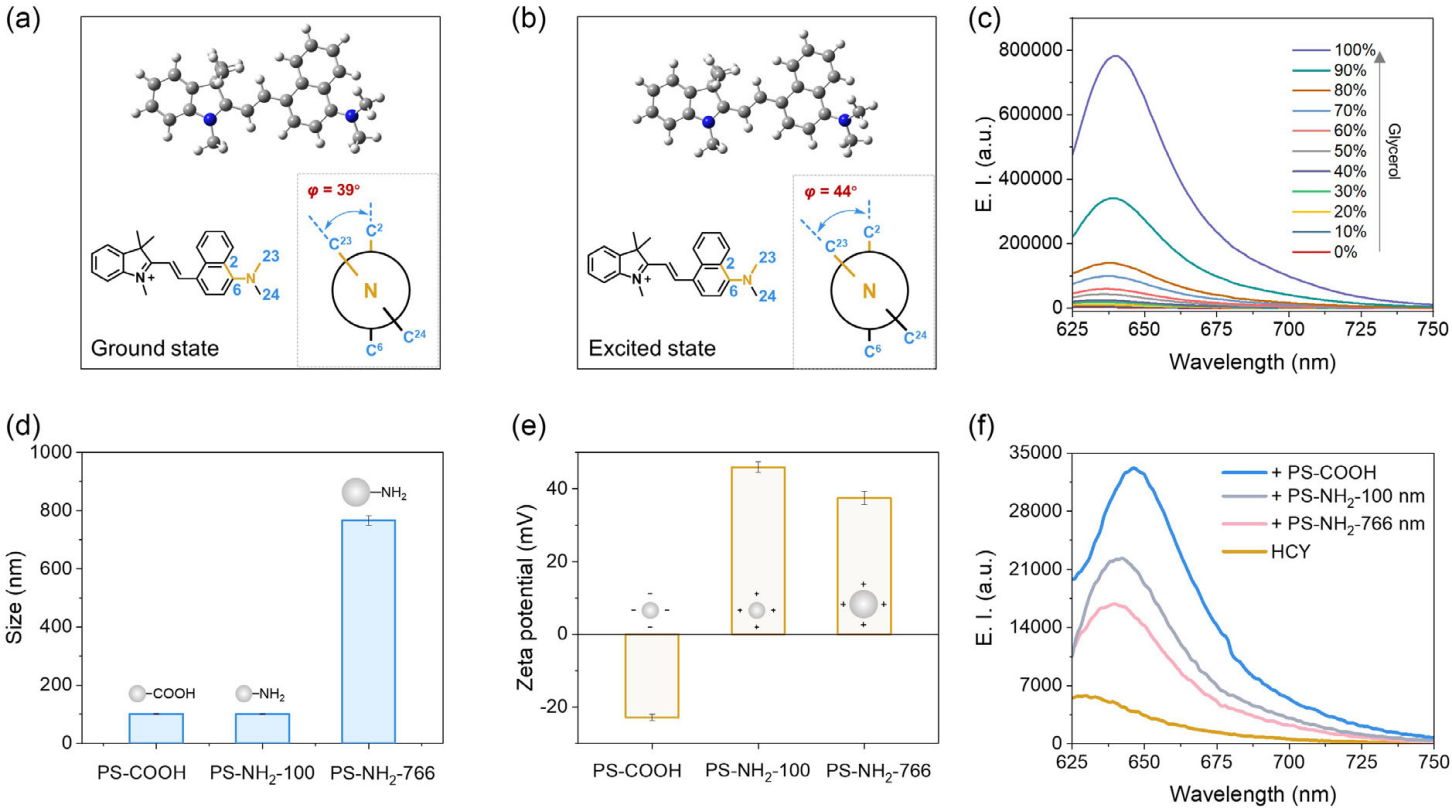
Detection mechanism of HCY. Minimum energy structures of HCY in a) the electronic ground state and b) the excited state calculated at the CAM‐B3LYP/cc‐pVDZ level. c) Emission profile of HCY in various volume ratios of water and glycerol. d) Particle size and e) zeta potential of PS‐COOH, PS‐NH_2_‐100, and PS‐NH_2_‐766 (mean ± SD, *n* = 3). f) Emission response of HCY to different PS nanoplastics. *λ*_ex_ = 615 nm; [HCY] = 5 μM; concentration of all nanoplastics is 100 μg mL^−1^.

Density functional theory (DFT) and time‐dependent density functional theory (TDDFT) calculations were performed to better understand the fluorescent response mechanism of HCY. The calculations were conducted using Gaussian 16, Revision A.03.^[^ ^29^ ^]^ The minimum‐energy structures of HCY and its dihedral angle *φ* were obtained using the CAM‐B3LYP functional^[^ ^30^, ^31^ ^]^ with the D3 version of Grimm's dispersion^[^ ^32^ ^]^ and the cc‐pVDZ basis sets.^[^ ^33^ ^]^ The dihedral angle *φ* of HCY changed from 39° in the ground state (**Figure** **3**a) to 44° in the excited state (Figure 3b), indicating that the naphthalene ring and the dimethylamino structure twist upon excitation. This torsional change suggests that the excited state of HCY can be characterized as a combination of a locally excited state (LE) and a twisted intramolecular charge transfer (TICT) state.^[^ ^34^, ^35^ ^]^ For fluorescent probes featuring a TICT mechanism, analytes binding typically alters the fluorescence intensity and wavelength, which is consistent with our observations (Figure 2d). Additionally, we calculated the HOMO/LUMO energy levels of HCY in different solvents using the B3LYP/6‐311 G(d,p) level of theory.^[^ ^36^, ^37^ ^]^ From the frontier molecular orbitals, the HOMO‐LUMO gap of HCY was 2.56 eV in aqueous solution and 2.54 eV in glycerol (Figure S8, Supporting Information). The smaller HOMO‐LUMO gap in glycerol indicates that the molecule is more prone to electron transfer in high‐viscosity solvents. This result aligns with the experimental observations (Figure 2a,e).

To evaluate whether HCY responds to viscosity, we measured its fluorescence spectra in an aqueous solution with varying glycerol concentrations. As the glycerol concentration increased, resulting in higher viscosity, the fluorescence intensity of HCY gradually enhanced (Figure 3c, Figure S9, Supporting Information). This enhancement is attributed to the restricted intramolecular motion of HCY in a more viscous environment, which suppresses nonradiative transitions and thereby strengthens the fluorescence signal. The viscosity values of 0% and 10% glycerol at 25 °C are 0.893 cP and 1.221 cP, respectively.^[^ ^38^ ^]^ An obvious HCY fluorescence change (Figure S8, Supporting Information) indicates that HCY is capable of detecting a viscosity change as low as 0.328 cP. To further verify the viscosity response, we tested the response in a mixture of water and DMSO. Similarly, an increase in fluorescence intensity with higher dimethyl sulfoxide (DMSO) concentrations was demonstrated, consistent with the increase in solution viscosity (Figure S10, Supporting Information).

To further confirm that the fluorescence enhancement originates from the viscous nanoenvironment surrounding nanoplastics rather than the surface charge, we tested the positively charged amino‐functionalized PS nanoplastics (PS‐NH_2_‐100) and bare PS nanoplastics (PS‐bare). The PS‐NH_2_‐100 nanoplastics (101 nm diameter) were similar in size to PS‐COOH (Figure 3d). Unlike the negatively charged PS‐COOH, PS‐NH_2_‐100 exhibited a positively charged surface, as confirmed by zeta potential measurements (Figure 3e). In the presence of the PS‐NH_2_‐100, HCY exhibited a 5.2‐fold fluorescence enhancement (Figure 3f, Figure S11, Supporting Information). We also tested larger‐sized amino‐functionalized PS nanoplastics (PS‐NH_2_‐766) with a diameter of 766 nm. Despite the larger particles having a relatively smaller surface area, a satisfactory fluorescence enhancement of HCY was observed (Figure 3f, Figure S12, Supporting Information). The fluorescence response was also confirmed with PS‐bare (Figure S13, Supporting Information), which lacks surface functional groups, showing that HCY detects nanoplastics by probing the viscous nanoenvironment surrounding them, regardless of their surface modification. We also extended the evaluation to other types of nanoplastics (PET and PP nanoplastics). Compared to PS nanoplastics, the PET and PP nanoplastics exhibited similar shapes in SEM images (Figure S14, Supporting Information) and triggered the fluorescence response of HCY (Figure S15, Supporting Information). The interaction of HCY with PET/PP is primarily attributed to the restricted intramolecular motion of HCY in a hydrophobic interfacial layer, with a further contribution from *π*–*π* stacking to PET but not the nonaromatic PP nanoplastics, resulting in a relatively weaker fluorescence response observed for PP.

To further elucidate the interaction between HCY and nanoplastics, we analyzed the surface electrical properties, infrared light absorption, morphology, and particle size of the nanoplastics before and after their interaction with HCY in PBS solution (pH = 7.4). The relatively larger charge change, indicative of a strong interaction, was observed for PS‐COOH and PET upon HCY addition (Figure S16, Supporting Information). This is attributed to polymer‐dependent sensitivity. In contrast, PS‐NH_2_ exhibited a slight increase in zeta potential, likely due to the absence of significant electrostatic interaction with HCY. We speculate that the binding of HCY to PS‐NH_2_ occurs primarily through hydrophobic interactions and *π*–*π* stacking, which aligns with fluorescence response exerted by HCY. FTIR analysis revealed two new peaks at 1282 and 752 cm^−1^ after PS‐COOH interacted with HCY‐PS (Figure S17, Supporting Information). The peaks correspond to the stretching vibration of C—N bond and the out‐of‐plane bending vibration of C—H bond in the naphthalene‐based donor rings of the HCY molecule. The appearance of these distinct peaks confirmed the attachment of HCY to the PS‐COOH surface. To examine whether the fluorescence response was caused by fluorophore‐induced aggregation, we measured the dynamic light scattering profiles of nanoplastics before and after the addition of HCY. The results showed no significant change in hydrodynamic size (Figure S18, Supporting Information), suggesting HCY does not induce nanoplastics aggregation. Properties of all nanoplastics used in the study are summarized and provided in Table S2, Supporting Information. Finally, we labeled PS‐COOH with HCY, removed unbound HCY by centrifugation, and captured images of the labeled PS‐COOH using confocal microscope (Figure S19, Supporting Information). The labeled PS‐COOH exhibited red fluorescence, providing further evidence that HCY effectively binds to PS‐COOH.

### 2.4 Live‐Cell Fluorescence Imaging of PS‐COOH

**Figure 4:**
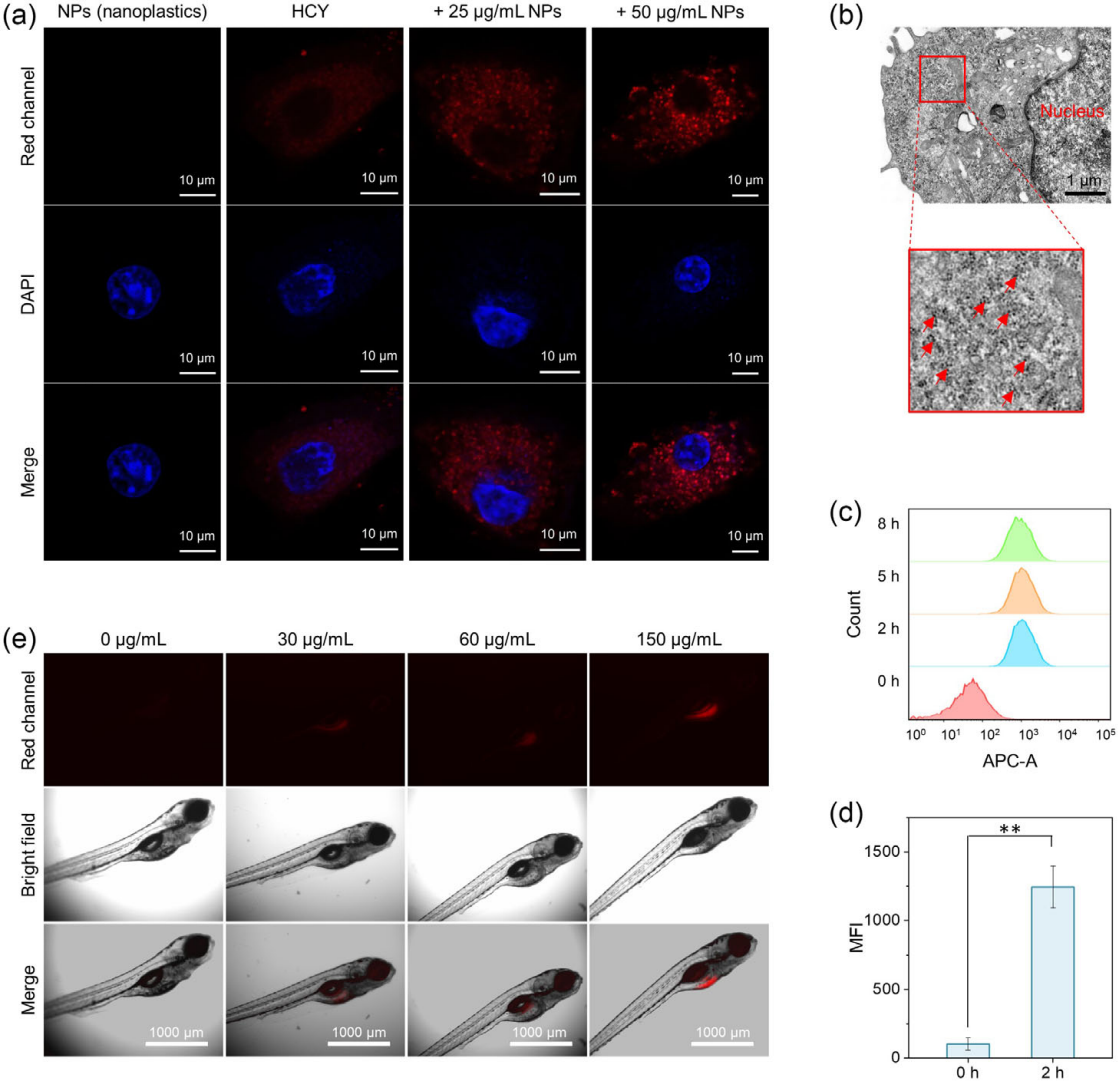
Fluorescence imaging in biological models. a) Live‐cell imaging of PS‐COOH by HCY in A549 cells. After preincubation with PS‐COOH for 24 h, the cells were treated with HCY (10 μM) for 2 h, fixed with 4% polyoxymethylene for 10 min, and costained with the nuclear staining dye 4′,6‐diamidino‐2‐phenylindole dihydrochloride (DAPI) for 10 min. DAPI was used to mark the location of cell nuclei. b) Bio‐TEM image of HepG2 cells treated with 100 μg mL^−1^ PS‐COOH for 24 h. c) Time‐dependent cellular uptake of nanoplastics using 200 μg mL^−1^ HL‐PS‐COOH by HepG2 cells via flow cytometry analysis. d) Mean fluorescence intensity in flow cytometric analysis (means ± SD, *n* = 3, ** *P* < 0.01). e) Fluorescence imaging of zebrafish cultured with HL‐PS‐COOH for 30 h. [HL‐PS‐COOH] = 0, 30, 60, and 150 μg mL^−1^.

To investigate the cellular uptake of HCY and determine whether it interacts with the cell membrane, we first performed the colocalization experiment using Dio and Lyso‐Tracker green. The results showed that HCY was efficiently taken up by cells and localized in lysosomes (Figure S20, Supporting Information). We next accessed the cytotoxicity of HCY via the 3‐(4,5‐dimethylthiazol‐2‐yl)‐2,5‐diphenyltetrazolium bromide (MTT) assay. HCY exhibited negligible cytotoxicity in both A549 and HepG2 cells at a concentration of 10 μM, confirming its good biocompatibility (Figure S21, Supporting Information). Confocal microscopy images of A549 cells incubated with nanoplastics alone exhibited no detectable fluorescence. In contrast, incubation with HCY showed a weak red fluorescence, which increased progressively with higher concentrations of PS‐COOH (**Figure** **4**a), indicating the effective detection of nanoplastics by HCY in live cells. To confirm that the enhanced fluorescence signals were specifically associated with PS‐COOH, we examined the cellular uptake of PS‐COOH nanoplastics using bio‐transmission electron microscopy (Bio‐TEM). Following incubation, PS‐COOH particles were observed to accumulate primarily in the cytoplasm (Figure 4b). Taken together, these findings highlight the ability of HCY to effectively label and image PS‐COOH in live cells, providing a valuable tool for studying nanoplastics and their interactions with biological systems.

### 2.5 Cellular Uptake Studies of PS‐COOH

Based on the excellent live‐cell imaging capability of HCY for PS‐COOH, we used HepG2 and A549 cells as model systems to investigate the cellular uptake of nanoplastics at varying exposure concentrations. Dye‐labeled nanoplastics have been frequently employed to study the uptake and biodistribution of nanoplastics.^[^ ^39^, ^40^, ^41^ ^]^ We first examined the time‐dependent uptake dynamics using the HCY‐labeled PS nanoplastics (termed HL‐PS‐COOH). The fluorescence intensity of the cells increased over time, reaching a plateau after 2 h of incubation with HL‐PS‐COOH, with no further increase observed thereafter. These findings suggest that cellular uptake of HL‐PS‐COOH reaches its maximum at 2 h (Figure 4c). Confocal imaging of HL‐PS‐COOH was then conducted to visualize the uptake of nanoplastics by living cells under exposure conditions. The imaging results of A549 cells coincubated with different concentrations of HL‐PS‐COOH clearly demonstrated a dose‐dependent intracellular accumulation (Figure S22, Supporting Information). These results indicate the capability of HL‐PS‐COOH for the further investigation of uptake, accumulation, and distribution of nanoplastics in vivo.

### 2.6 Fluorescence Imaging of PS‐COOH in Zebrafish

Nanoplastics are readily ingested by aquatic animals, leading to toxic effects. Zebrafish are widely used as a biological model for evaluating the toxicity of environmental contaminants, with different developmental stages providing valuable insights into the health risks of nanoplastics. Due to their transparency, zebrafish larvae are particularly suitable for real‐time imaging, allowing the direct visualization of fluorescently labeled nanoplastics. As shown in Figure 4e and Figure S23, Supporting Information, the accumulation of HL‐PS‐COOH in zebrafish increased gradually with the rising concentrations of HL‐PS‐COOH. Notably, the imaging results indicated that HL‐PS‐COOH primarily accumulated in the yolk sac, which is consistent with previously reported findings.^[^ ^42^ ^]^

## 3 Conclusions

In summary, we develop a novel strategy to design fluorescent probes for nanoplastics and demonstrate their applicability across several types of model nanoplastics. The designed probe, HCY, exhibits a rapid and sensitive detection by probing the viscous nanoenvironment surrounding them. HCY binds to nanoplastics mainly through restricted intramolecular motion, with electrostatic adsorption modulating only the negatively charged nanoplastics and *π*–*π* stacking exclusively to those containing aromatic rings. The viscous nanoenvironment on the nanoplastics surface inhibits the TICT process of HCY, producing a largely enhanced fluorescence signal and leading to a low detection limit. Additionally, HCY demonstrates excellent potential for live‐cell imaging of the PS‐COOH. Using HCY as a fluorescent probe, we labeled PS‐COOH and successfully visualized the uptake and biodistribution in live cells as well as in zebrafish models. As a proof of concept, this study focuses on demonstrating our new sensing strategy using these well‐established model nanoplastics (i.e., PS‐based particles). In real‐world applications, nanoplastics may vary in shape and morphology. However, we anticipate that the probe will still remain responsive, as the sensing mechanism relies on detecting the viscous nanoenvironment surrounding the nanoplastics.

## 4 Experimental Section

4.1

4.1.1

##### General Information of Materials and Instruments

Unless otherwise noted, all reagents and solvents were obtained from commercial suppliers and used without further purification. PS‐COOH, PS‐NH_2_‐766, and PS‐bare nanoplastics were ordered from Macklin Biochemical Technology Co., Ltd. (Shanghai, China). PS‐NH_2_‐100 nanoplastics were ordered from Yuan Biotech Co., Ltd. (Shanghai, China). PET and PP nanoplastics were ordered from Zhongkeleiming Daojin Technology Co., Ltd. (Beijing, China). Cell membrane green fluorescent probe Dio and lysosomal green fluorescent probe Lyso‐Tracker Green were ordered from Beyotime Biotechnology Co., Ltd. (Shanghai, China). Wild‐type zebrafish embryos were ordered from Shanghai FishBio Co., Ltd. (Shanghai, China).

Thin‐layer chromatography was performed using precoated silica gel 60 F254 aluminum sheets. Column chromatography was conducted using silica gel and laboratory grade solvents manually. ^1^H NMR and ^13^C NMR were recorded on Qone Quantum‐I‐Plus 400 MHz and Varian Mercury plus 400 MHz NMR spectrometer, respectively. High‐resolution mass spectrum was recorded on Thermo Scientific Q Exactive Orbitrap Mass Spectrometer. SEM image was acquired by SIGMA HD (Zeiss, Germany) and SU8600 (HITACHI, Japan). Bio‐TEM image was acquired by HT7700 (HITACHI, Japan). Absorption spectrum was measured on SPECORD 210 PLUS UV/VIS spectrophotometer (Analytic Jena, Germany). Fluorescence spectrum was measured on FLS1000 steady‐state and transient fluorescence spectrometer (Edinburgh, UK). Cytotoxicity assay was measured by BioTek Synergy‐2 fully automated microplate reader. Cell imaging experiment was performed using ZEISS LSM 980 with Airyscan 2 confocal laser scanning microscope. Flow cytometric analysis was obtained from BD FACSVerse (Becton, Dickinson and Company, USA). Zebrafish imaging experiment was performed using ThermoFisher Invitrogen EVOS FL Auto Cell Imaging System.

##### Synthesis

The probe HCY was synthesized as described in Scheme S1, Supporting Information, with characterization data (including ^1^H NMR, ^13^C NMR, and ESI‐MS spectra) provided in Figures S1–S3, Supporting Information.

##### DFT and TDDFT Calculations

DFT and TDDFT calculations were performed using Gaussian 16, Revision A.03.^[^ ^29^ ^]^ The minimum energy structure of HCY and dihedral angle φ of HCY were obtained using the CAM‐B3LYP functional^[^ ^30^, ^31^ ^]^ and the D3 version of Grimm's dispersion^[^ ^32^ ^]^ with the cc‐pVDZ basis sets.^[^ ^33^ ^]^ The solvent effect was neglected in these calculations. HOMO/LUMO energy level calculations of HCY in different solvents were performed at the B3LYP/6‐311 G(d,p) level^[^ ^36^, ^37^ ^]^ under the SMD solvent model.^[^ ^43^ ^]^ The calculated results were analyzed and visualized by Multiwfn Version 3.8(dev),^[^ ^44^ ^]^ and VMD Version 1.9.3.^[^ ^45^ ^]^ Geometry optimization and vibrational analysis were performed at the same level (number of imaginary frequencies is 0 for minima).

##### Photophysical Measurements

All spectra were measured using a stock solution of the probes (4 mM in DMSO), which was diluted with pH 7.4 PBS buffer to the desired concentration. During the test, the solution was placed in a quartz cuvette with an optical path of 1 cm. Luminescence titration was performed by measuring emission of HCY in pH 7.4 PBS buffer with the gradually increased concentration of nanoparticles. Stability was evaluated by measuring emission of HCY or HCY + nanoplastics in pH 7.4 PBS buffer as a function of time. pH sensitivity was performed by measuring emission of HCY or HCY + nanoplastics in aqueous solution with varying pH conditions. Aqueous solution with different pH value was prepared by adding 1 M HCl or NaOH to deionized water. Selectivity assay was performed by measuring emission of HCY in pH 7.4 PBS buffer in presence of varying analytes including typical ions, amino acids, biological macromolecules, and hydrophobic particulates commonly existed in living organisms, as well as PS‐COOH.

##### Determination of Detection Limit

The detection limit of HCY for PS‐COOH based on the IUPAC definition (signal‐to‐noise ratio S/N = 3) is calculated by the linear function and the following equation

(1) Detection limit = 3σ/k

where *σ* is the standard derivation of emission experiments of blank solutions; and *k* is the slope of the linear calibration curve.

##### Cell Culture

HepG2 and A549 cells were used in this study. HepG2 cells were cultured in dulbecco's modified eagle medium (DMEM)‐F12 supplemented with 15% fetal bovine serum, 1% penicillin, and 1% streptomycin in a CO_2_ incubator at 37 °C. A549 cells were cultured in high‐glucose DMEM supplemented with 10% fetal bovine serum, 1% penicillin, and 1% streptomycin in a CO_2_ incubator at 37 °C.

##### Cytotoxicity Assay

Cytotoxicity of HCY was evaluated by MTT assay. Briefly, 6 × 10^3^ cells/100 μL per well were seeded in 96‐well plates and incubated for 24 h in a CO_2_ incubator at 37 °C. HCY at different concentrations were added to the cells. After overnight incubation, 10 μL of MTT solution (5 mg mL^−1^) was added to each well, and the cells were incubated for another 4 h. Then, 100 μL of DMSO was added to dissolve the formed formazan crystals. After shaking for 10 min, absorbance was read at 490 nm using a BioTek Synergy‐2 fully automated microplate reader. Viability of the cells without the probe is defined as 100%.

##### Live‐Cell Fluorescence Imaging

HepG2 cells (8 × 10^3^ cells per well) were seeded into a confocal dish and incubated in a CO_2_ incubator at 37 °C for 24 h. Cells were treated with PS‐COOH at different concentrations (0, 20, 50, and 100 μg mL^−1^). After 24 h of incubation, 10 μM HCY was added and incubated with the cells for 2 h. The cells were then fixed with 4% polyoxymethylene for 10 min. Afterward, the nuclei were stained using DAPI for 10 min. Fluorescence images were measured by ZEISS LSM 980 with Airyscan 2 confocal laser scanning microscope.

##### Colocalization Experiment

A549 cells (8 × 10^3^ cells per well) were seeded into a confocal dish and incubated in a CO_2_ incubator at 37 °C for 24 h. After incubation with HCY (10 μM) for 2 h, cells were costained with cell membrane green fluorescent probe Dio for 10 min. Fluorescence images were measured by ZEISS LSM 980 with Airyscan 2 confocal laser scanning microscope.

##### Flow Cytometry Analysis

HepG2 cells were seeded into 12‐well plates (1 × 10^5^ cells/well) and incubated overnight to achieve adherence. The cells were exposed to HL‐PS‐COOH (200 μg mL^−1^) for varying durations (0, 2, 5, 8 h), or an equal volume of PBS for 8 h. Then, the cells were washed twice with cold PBS and harvested by trypsinization, followed by centrifugation (1500 *x*g, 5 min). The obtained cells were resuspended in PBS and analyzed using a flow cytometer (BD FACSVerse). 10 000 events were acquired with three replicates for each treatment condition. Gating was carried out as follows: A. Forward (FSC‐Area) versus side scatter (SSC‐Area); B. FSC‐Area/FSC‐Height (to exclude doublets); and C. Analyzed for APC signal area. All instrument settings were kept fixed during operation.

##### Fluorescence Imaging of Zebrafish

Zebrafish were cultured in E3 embryo media (15 mM NaCl, 0.5 mM KCl, 1 mM MgSO_4_, 1 mM CaCl_2_, 0.15 mM KH_2_PO_4_, 0.05 mM Na_2_HPO_4_, and 0.7 mM NaHCO_3_) under a water circulation system at 28 °C and a 14 h light and 10 h dark cycle. For the imaging experiment, three‐day‐old zebrafish larvae were randomly allocated into four groups (6 larvae per group) and exposed to 0, 30, 60, and 150 μg mL^−1^ HL‐PS‐COOH, respectively. The culture medium was supplemented with 1‐phenyl‐2‐thiourea (200 μM) to maintain the transparency of the zebrafish larvae. After 30 h of exposure, the larvae were washed with PBS, anesthetized with MS‐222 (100 mg L^−1^), and imaged using Thermo Fisher Scientific Invitrogen EVOS FL Auto Cell Imaging System. Each group of tests was performed in triplicate. The experiment was performed in compliance with the relevant laws and institutional guidelines and was approved by the Animal Ethical Experimentation Committee of Central China Normal University (CCNU).

##### Statistical Analysis

All measurements in this study were repeated three times. Data were presented as mean ± SD. Statistical significance between two groups was analyzed using the single‐sample T‐test method (two‐tailed; * *P* < 0.05, ** *P* < 0.01, and *** *P* < 0.001).

## Supporting Information

Supporting Information is available from the Wiley Online Library or from the author.

## Conflict of Interest

The authors declare no conflict of interest.

## Supporting information

Supplementary Material

## Data Availability

The data that support the findings of this study are available from the corresponding author upon reasonable request.
