# Supplementary material for: Identification of Nanoplastics by Probing the Viscous Nanoenvironment
Source: Small Sci. 2025 Nov 10;5(12):e202500430. doi: 10.1002/smsc.202500430 (PMC12697767; doi:10.1002/smsc.202500430)
Supplement: Supplementary file 1 — Supplementary Material [file SMSC-5-e202500430-s001.pdf]

Supplementary Information for

Identification of Nanoplastics by Probing the Viscous Nano-Environment

Liang Li,#,[a] Wenjie Yang, #,[a] Yonggen Hong,[a] Qiyuan He,[a] Xuanyi Lu,[a] Hong Wang,[a] Peng Tao,[b] Chao Shu,*[c] Mingqing Chen,*[a] Guochen Bao,*[d] and Lijun Jiang*[a]

[a] Key Laboratory of Pesticide and Chemical Biology of Ministry of Education, Hubei Key Laboratory of Genetic Regulation & Integrative Biology, School of Life Sciences, Central China Normal University, Wuhan 430079, China

[b] Department of Applied Biology and Chemical Technology, The Hong Kong Polytechnic University, Hong Kong SAR, China

[c] State Key Laboratory of Green Pesticide, College of Chemistry, Central China Normal University, Wuhan 430079, China

[d] School of Mathematical and Physical Sciences, Faculty of Science, University of Technology Sydney, Sydney, New South Wales, 2007, Australia

# These authors contributed equally to this work.

E-mail: lijunjiang@ccnu.edu.cn, guochen.bao@uts.edu.au, chenmq@ccnu.edu.cn, chaoshu@ccnu.edu.cn

1. Synthesis and Characterization

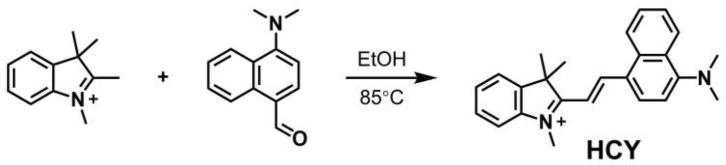

Scheme S1. The synthetic route towards **HCY**.

4-Dimethylamino-1-naphthaldehyde (100 mg, 0.50 mmol) and 1,2,3,3-Tetramethyl-3H-indolium iodide (152 mg, 0.50 mmol) were added to ethanol (10 mL). The mixed solution was heated to 85°C under an inert atmosphere of nitrogen with the protection from light. After stirred for 12 h, the mixture was cooled to room temperature and the solvent was removed under reduced pressure. Purification by silica gel column chromatography (CH_2Cl_2/MeOH) provided (*E*)-2-(2-(4-(dimethylamino)naphthalen-1-yl)vinyl)-1,3,3- trimethyl-3*H*-indol-1-ium (named HCY due to its structural similarity to hemicyanine dyes) as a tawny solid (160 mg, yield: 90 %).

^{1}H NMR (400 MHz, MeOD-*d*$_4$) δ 9.05 (d, *J* = 15.6 Hz, 1H), 8.52 (d, *J* = 8.6 Hz, 1H), 8.36 (d, *J* = 8.4 Hz, 1H), 8.29 – 8.19 (m, 1H), 7.79 – 7.69 (m, 3H), 7.64 – 7.53 (m, 4H), 7.21 (d, *J* = 8.6 Hz, 1H), 4.11 (s, 3H), 3.21 (s, 6H), 1.91 (s, 6H).

^{13}C NMR (101 MHz, DMSO-*d*$_6$) δ 180.07, 157.12, 147.88, 142.84, 142.07, 133.73, 130.49, 128.88, 128.61, 128.31, 126.17, 125.73, 125.26, 123.31, 122.79, 122.40, 114.30, 112.55, 109.45, 51.39, 44.28, 33.86, 26.40.

HRMS (m/z): calcd. for $C_{25}H_{27}N_2$ $[M]^+$ 355.2169, found 355.2163.

2. Supplementary figures

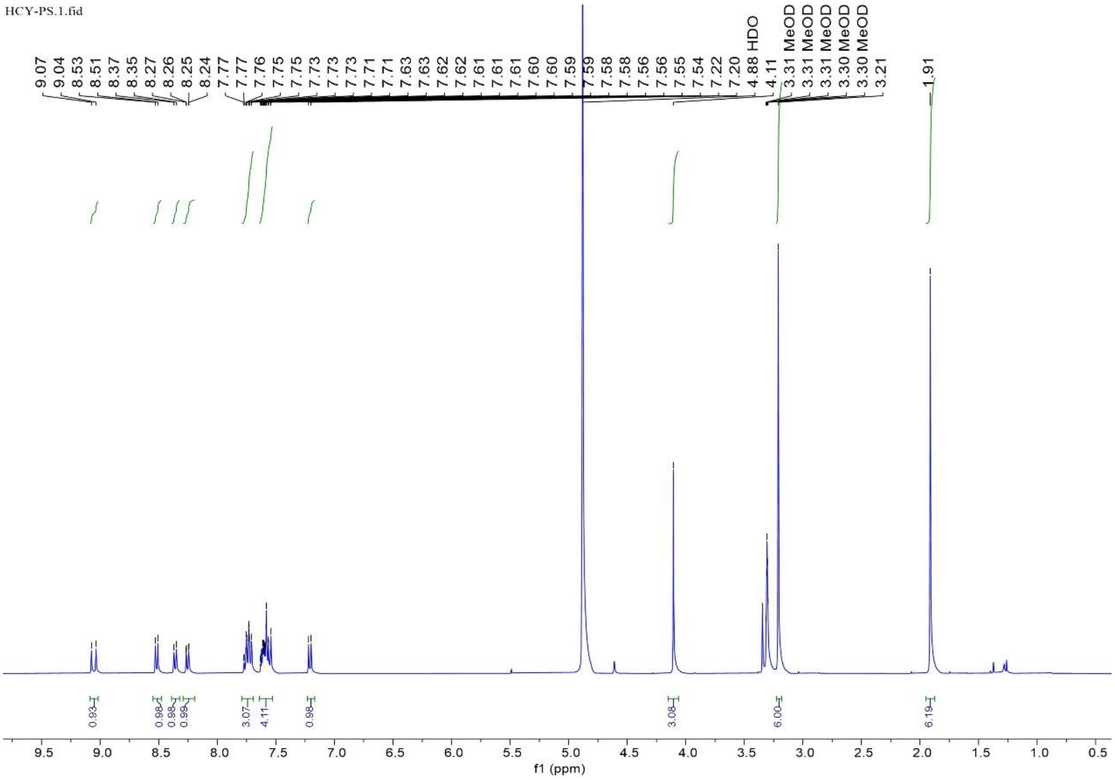

Figure S1. ^{1}H NMR spectrum of HCY (400 MHz, MeOD-*d*$_4$).

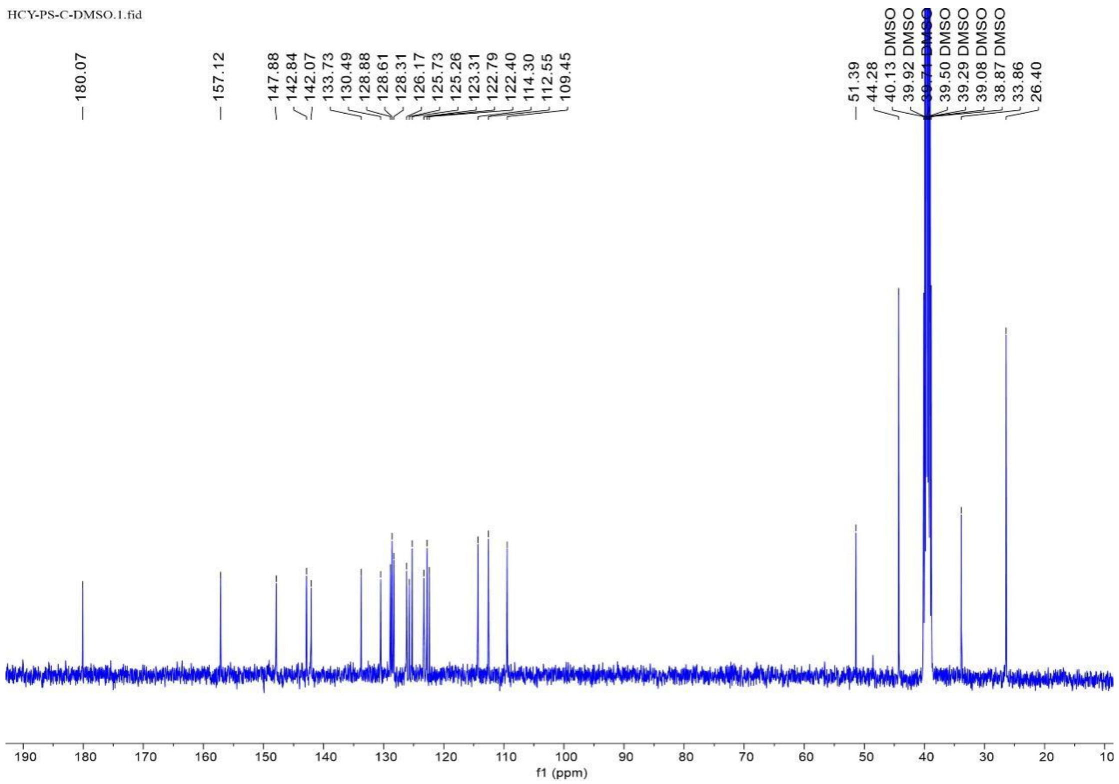

Figure S2. ^{13}C NMR spectrum of HCY (101 MHz, DMSO-d_6).

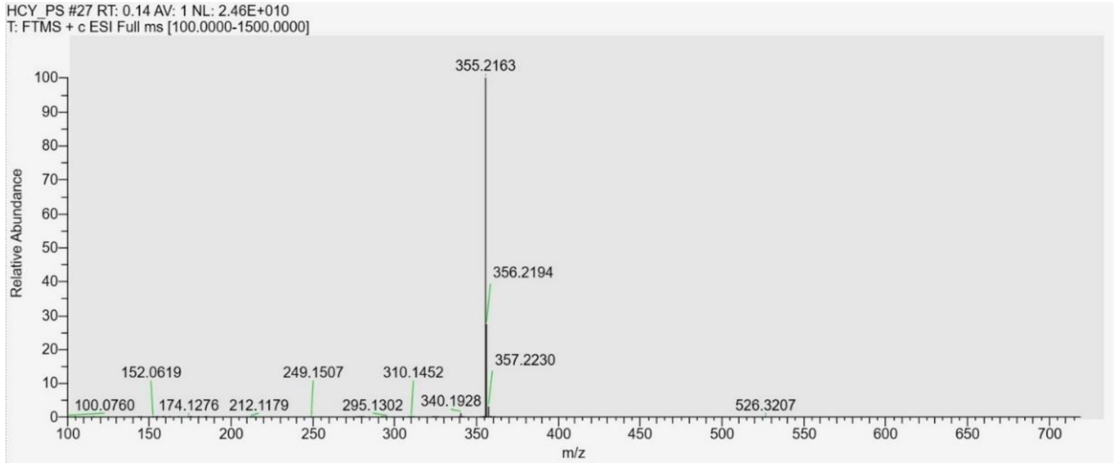

Figure S3. High-resolution mass spectrum of HCY.

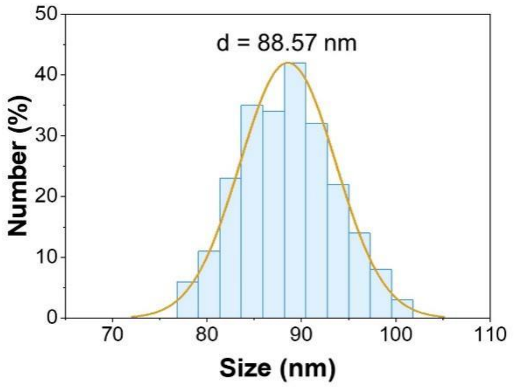

Figure S4. Particle size distribution of PS-COOH.

Table S1. Comparison with other methods for nanoplastics detection.

Type and size of nanoplastics	Method	Detection limit	Ref.
Polystyrene: 100 nm	Fluorometric	0.518 μg/mL	[1]
Polystyrene: 100, 300, 460, 600, 800 nm	Surface-enhanced raman scattering	0.31, 0.53, 0.76, 1.04, 1.42 μg/mL	[2]
Polystyrene: 100 nm	Fluorometric	10 μg/mL	[3]
Polystyrene: 200 nm	Laser-backscattering fiber-embedded optofluidic chip	0.23 μg/mL	[4]
Polystyrene: 70 nm	Electrochemical	1 μg/mL	[5]
Polypropylene: 230 nm	Colorimetric	9.95 μg/mL	[6]
Polypropylene: 100 nm	Electrochemiluminescence	0.948 μg/mL	[7]
Polystyrene: 130 nm	Surface-enhanced raman scattering	10 μg/mL	[8]
Polystyrene: 100 nm	Fluorometric	0.153 μg/mL	This work

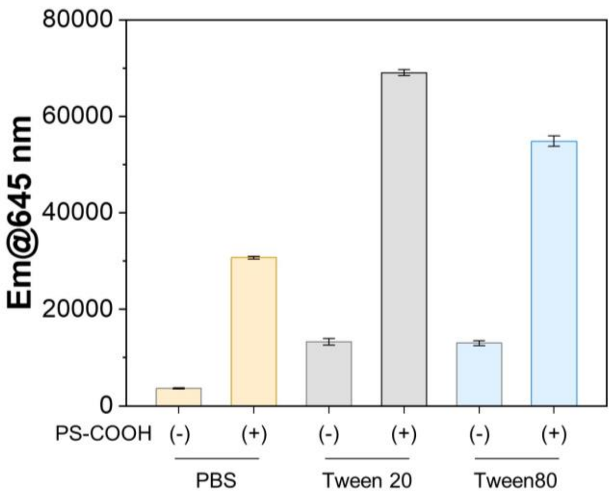

Figure S5. Emission intensity of HCY and HCY + PS-COOH in PBS, PBS containing 0.1% Tween 20, and PBS containing 0.1% Tween 80. λ_{ex} = 615 nm, [HCY] = 5 μM, [PS-COOH] = 100 μg/mL. Data are presented as mean ± SD (n = 3).

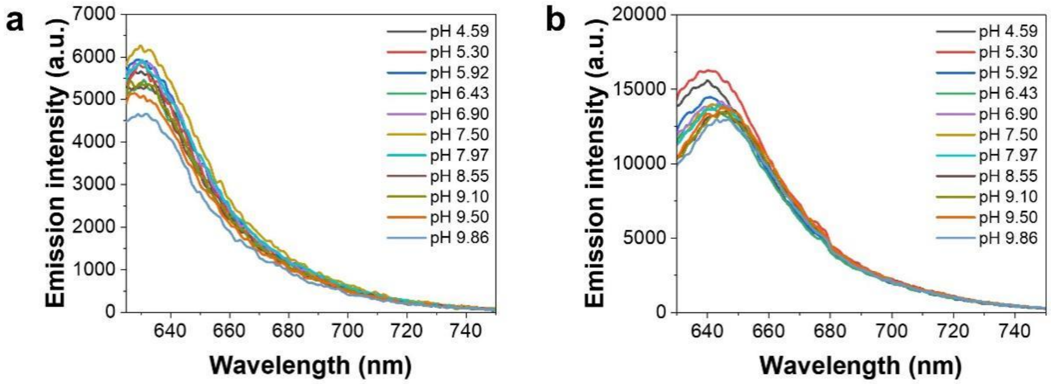

Figure S6. Emission profile of (a) HCY alone and (b) upon incubation with 40 μg/mL PS-COOH as a function of pH (4.5-10.0). λ_{ex} = 615 nm, [HCY] = 5 μM.

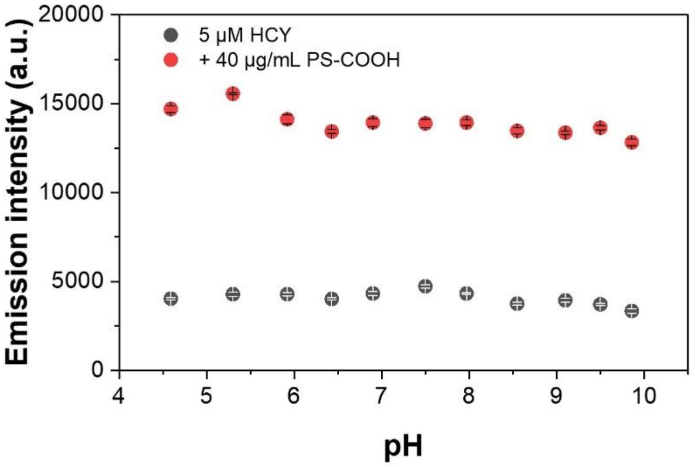

Figure S7. Emission intensity at 645 nm of HCY alone and upon addition of PS-COOH. λ_{ex} = 615 nm, [HCY] = 5 μM, [PS-COOH] = 40 μg/mL. Data are presented as mean ± SD (n = 3).

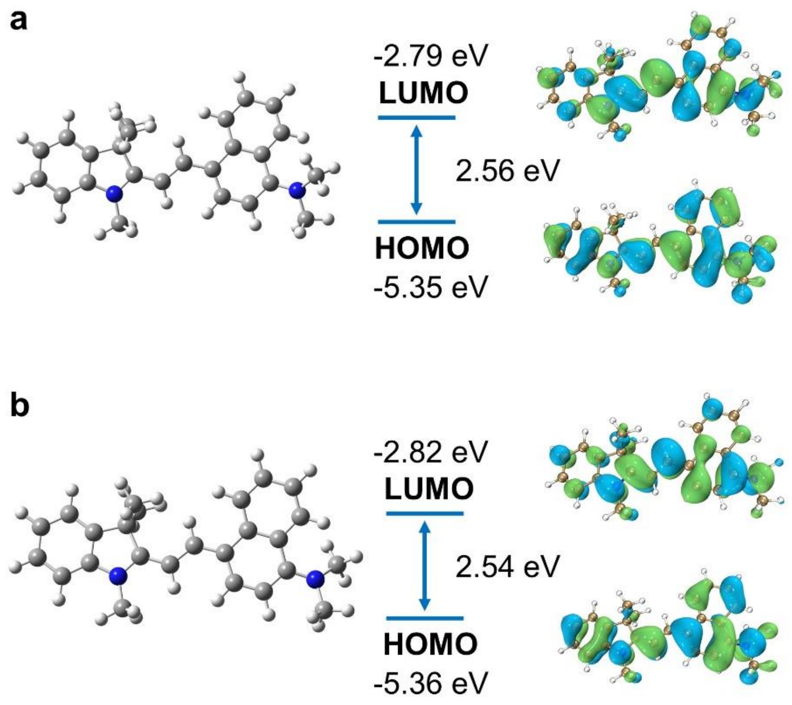

Figure S8. The ground state S_0 geometries and frontier molecular orbitals of HCY in (a) water and (b) glycerol.

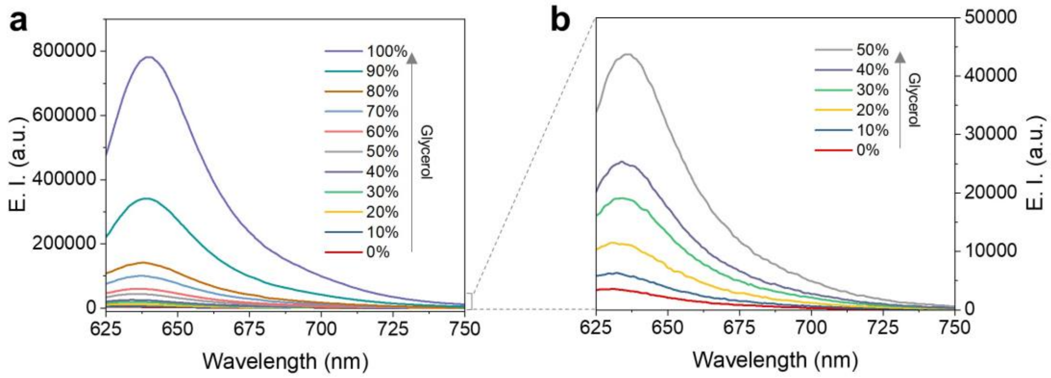

Figure S9. (a) Emission profile and (b) enlarged emission profile of HCY in various volume ratios of water and glycerol. λ_{ex} = 615 nm, [HCY] = 5 μM.

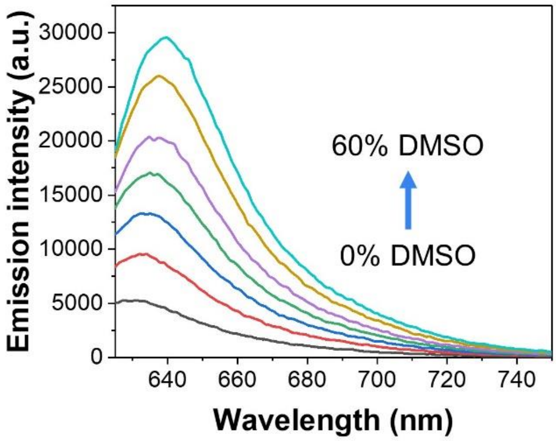

Figure S10. Emission profile of HCY in different water fractions of H_2O/DMSO mixed solvents. λ_{ex} = 615 nm, [HCY] = 5 µM.

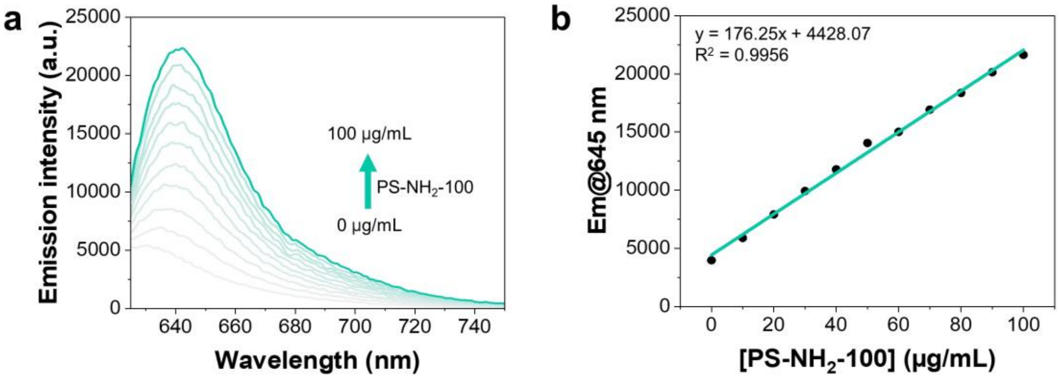

Figure S11. (a) Emission profile of HCY with PS-NH_2-100 ranging from 0-100 µg/mL. (b) Linearity between emission intensity of HCY at 645 nm and concentrations of PS-NH_2-100 from 0-100 µg/mL. λ_{ex} = 615 nm, [HCY] = 5 µM.

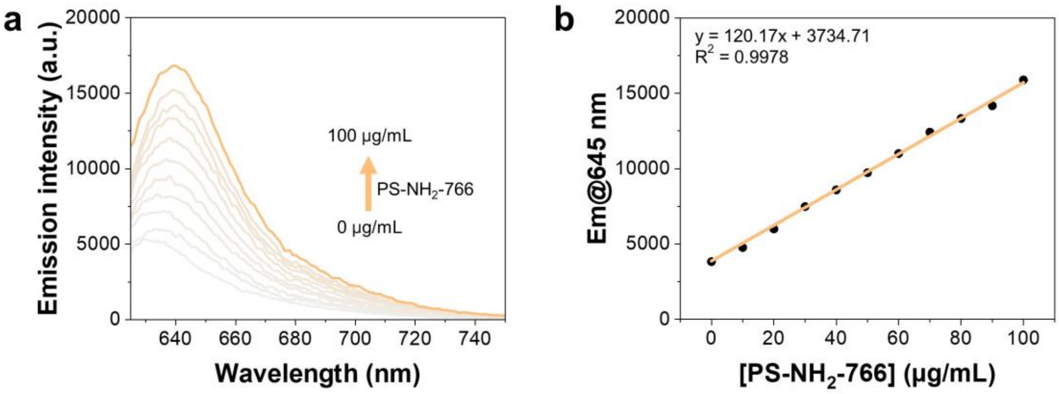

Figure S12. (a) Emission profile of HCY with PS-NH_2-766 ranging from 0-100 µg/mL. (b) Linearity between emission intensity of HCY at 645 nm and concentrations of PS-NH_2-766 from 0-100 µg/mL. λ_{ex} = 615 nm, [HCY] = 5 µM.

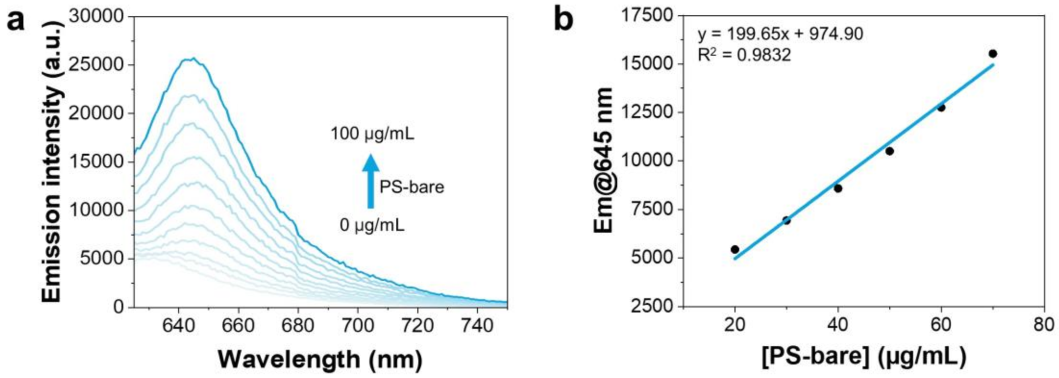

Figure S13. (a) Emission profile of HCY with PS-bare ranging from 0-100 μg/mL. (b) Linearity between emission intensity of HCY at 645 nm and concentrations of PS-bare from 20-70 μg/mL. λ_{ex} = 615 nm, [HCY] = 5 μM.

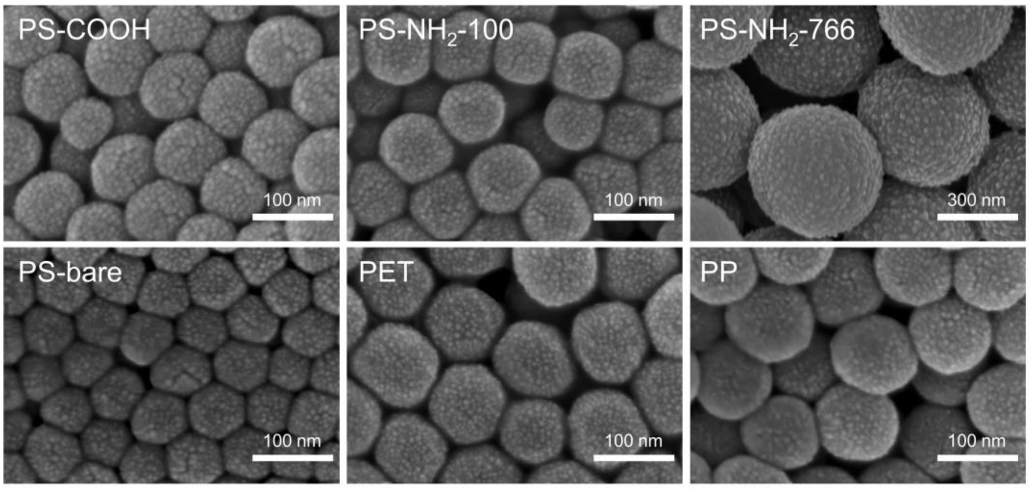

Figure S14. Scanning electron microscope (SEM) images of nanoplastics used in the study.

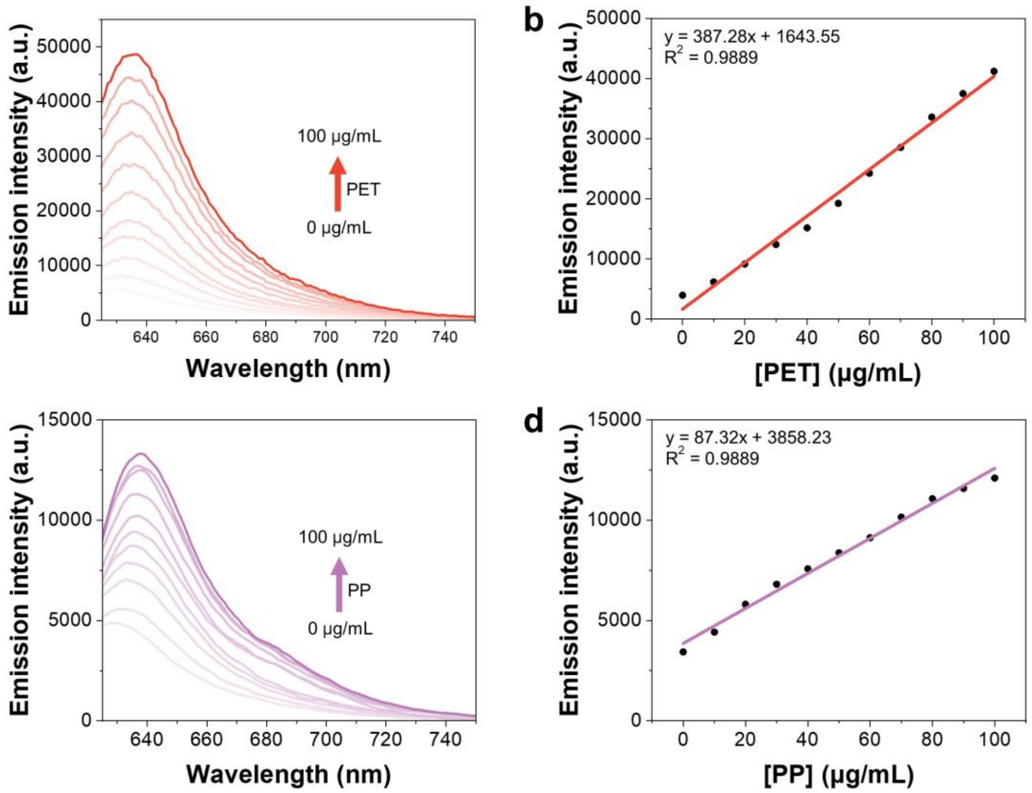

Figure S15. (a) Emission profile of HCY with PET ranging from 0-100 μg/mL. (b) Linearity between emission intensity of HCY at 645 nm and concentrations of PET from 0-100 μg/mL. (c) Emission profile of HCY with PP ranging from 0-100 μg/mL. (d) Linearity between emission intensity of HCY at 645 nm and concentrations of PP from 0-100 μg/mL. λ_{ex} = 615 nm, [HCY] = 5 μM.

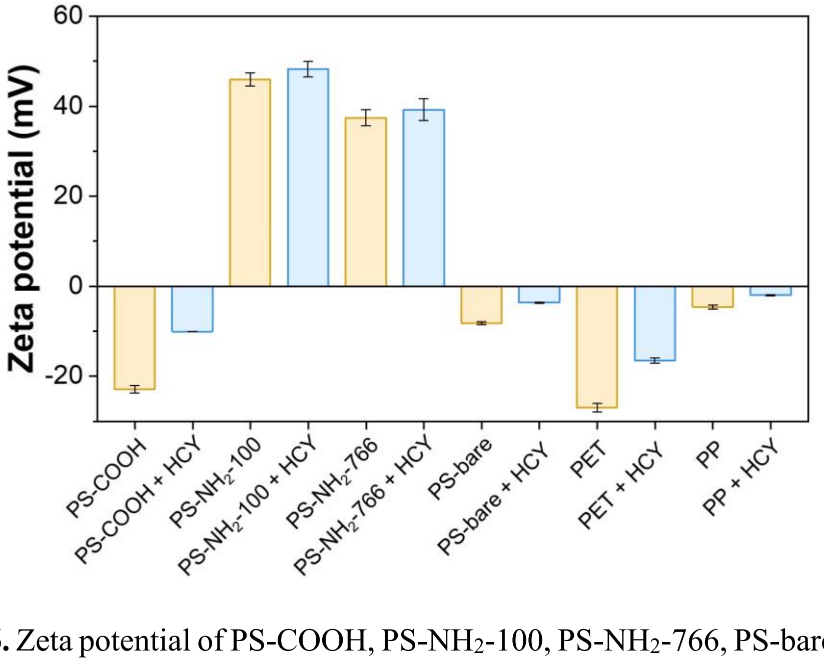

Figure S16. Zeta potential of PS-COOH, PS-NH_2-100, PS-NH_2-766, PS-bare, PET, and PP alone and with HCY. [HCY] = 5 μM, [nanoplastics] = 40 μg/mL. Data are presented as mean ± SD (n = 3).

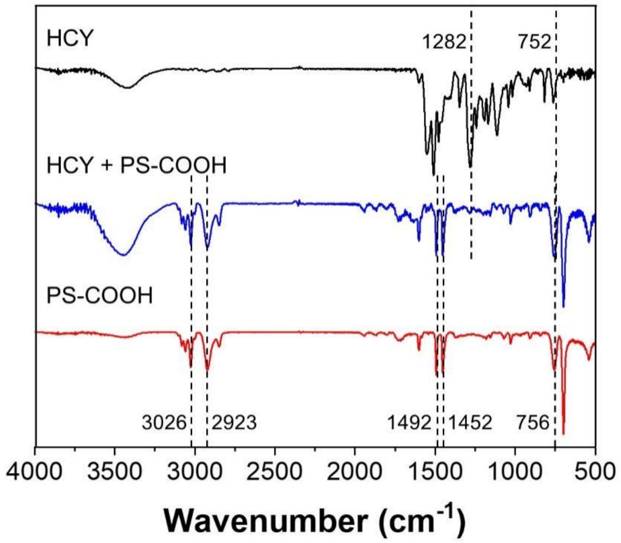

Figure S17. Fourier transform infrared spectrometer analysis of HCY, HCY + PS-COOH, and PS-COOH.

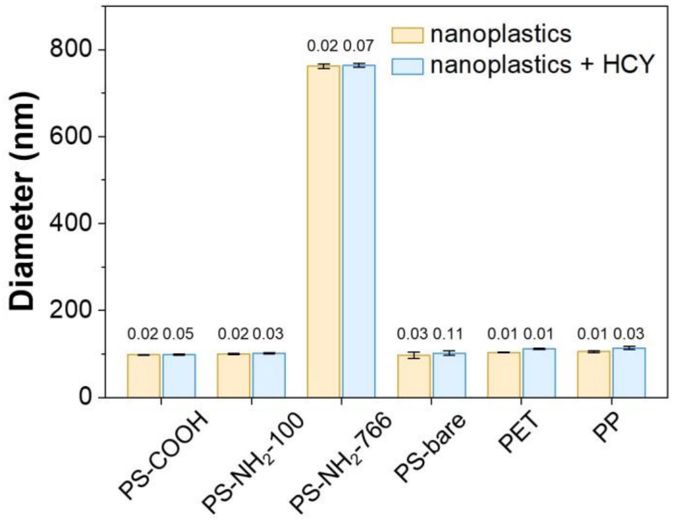

Figure S18. DLS measurements of size and PDI (number above bars) of PS-COOH, PS-NH_2-100, PS-NH_2-766, PS-bare, PET, PP alone and with HCY. [nanoplastics] = 40 µg/mL, [HCY] = 5 µM. Data are presented as mean ± SD (n = 3).

Table S2. Properties of the nanoplastics used in the study.

Type	Name	Average diameter [a]	Charge [b] and modification	Sources
Polystyrene	PS-COOH	101.65 nm	Anionic (-COOH)	L815962 [c]
	PS-NH_2-100	100.96 nm	Cationic (-NH_2)	YM1100A [d]
	PS-NH_2-766	766.46 nm	Cationic (-NH_2)	L861546 [c]
	PS-bare	97.12 nm	Anionic	L815936 [c]

Polyethylene terephthalate	PET	103.30 nm	Anionic	PET000100 [e]
Polypropylene	PP	105.39 nm	Anionic	PP000100 [e]

[a] Values are calculated from at least three independent measurements using dynamic light scattering.

[b] Charges are determined from at least three independent measurements using a Malvern Laser Particle Size Analyzer.

[c] Nanoplastics were purchased from Shanghai Macklin Biochemical Co., Ltd.

[d] Nanoplastics were purchased from Yuan Biotech Co., Ltd.

[e] Nanoplastics were purchased from Beijing Zhongkeleiming Daojin Technology Co., Ltd.

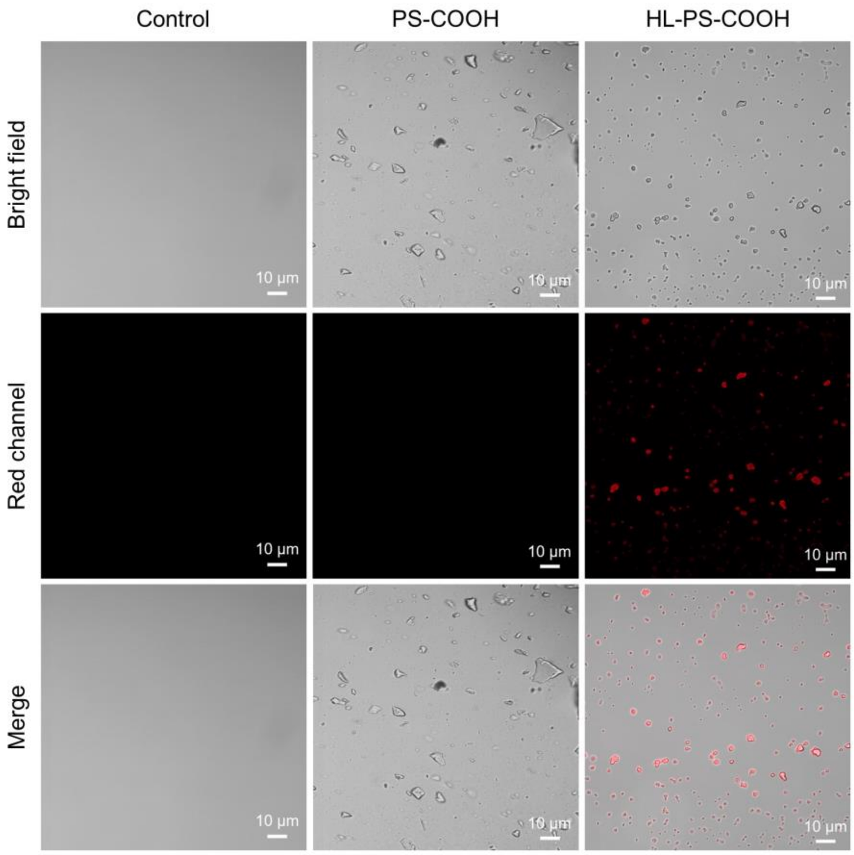

Figure S19. Laser scanning confocal microscope imaging of PS-COOH and HL-PS-COOH. The labeled HL-PS-COOH was obtained by freeze-drying and used directly for fluorescence imaging.

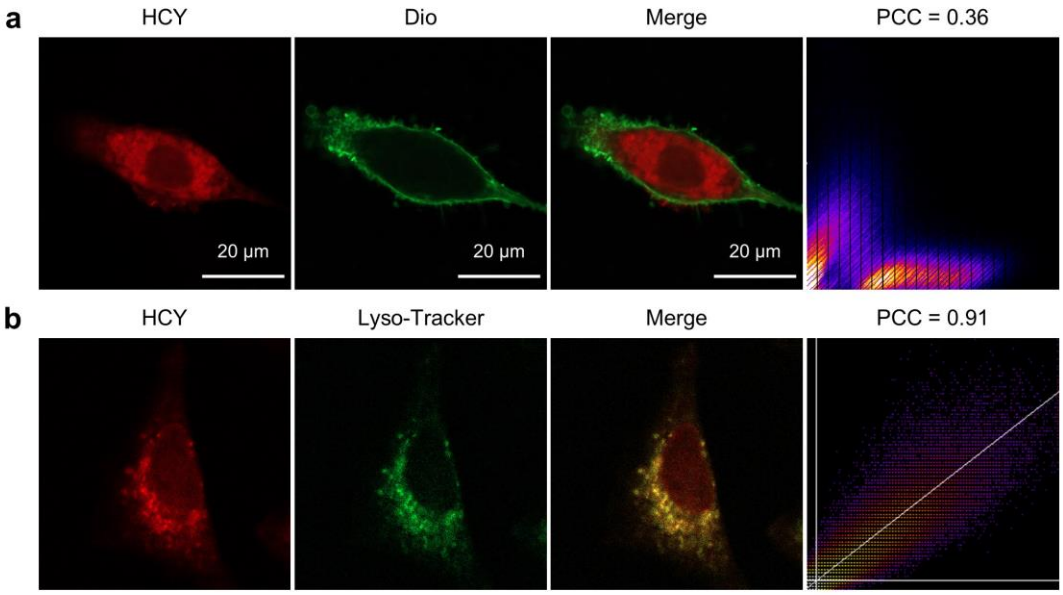

Figure S20. Colocalization of HCY and (a) Dio and (b) Lyso-Tracker in A549 cells. Cells incubated with HCY (10 μM) for 2 h and then co-stained with Dio or Lyso-Tracker for 10 min. Pearson's correlation coefficient was evaluated by ImageJ.

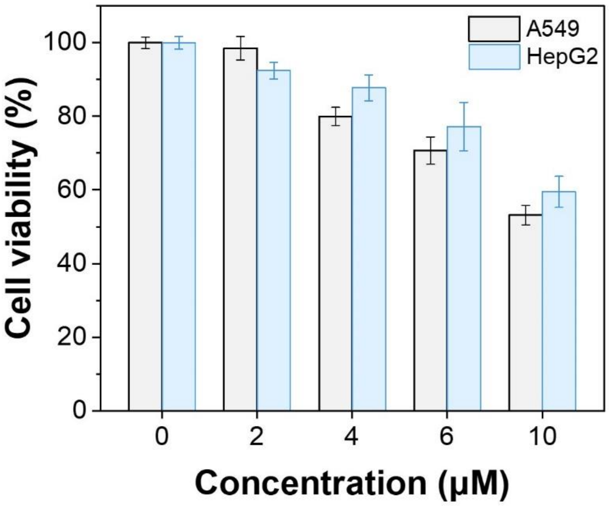

Figure S21. Cytotoxic effects of HCY on A549 and HepG2 cells. Data are presented as mean ± SD (n = 3).

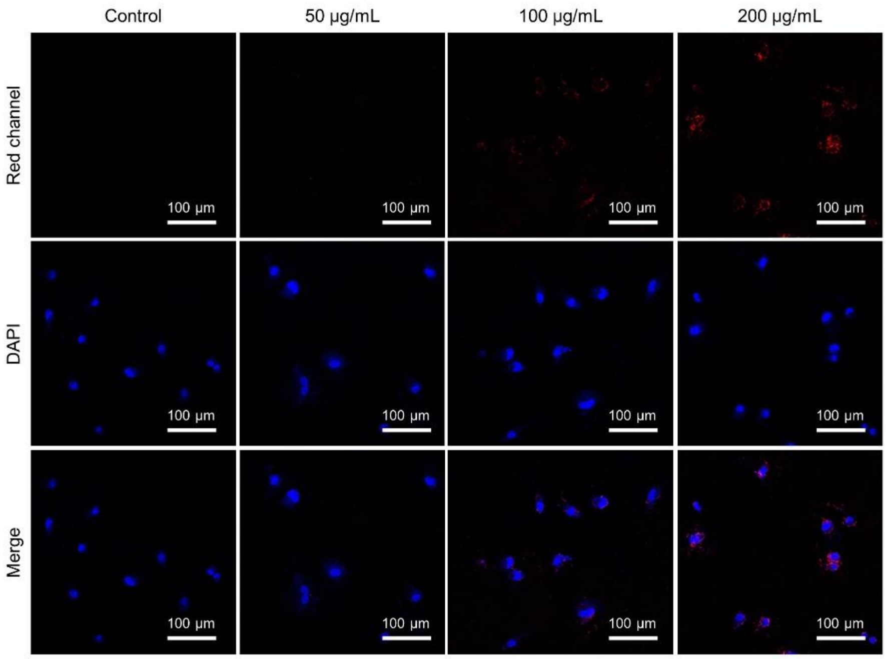

Figure S22. Live-cell imaging of HL-PS-COOH in A549 cells, for the study of cellular uptake of nanoplastics. The cells were treated with HL-PS-COOH for 2 h, fixed with 4% polyoxymethylene for 10 min, and co-stained with the nuclear staining dye DAPI for 10 min.

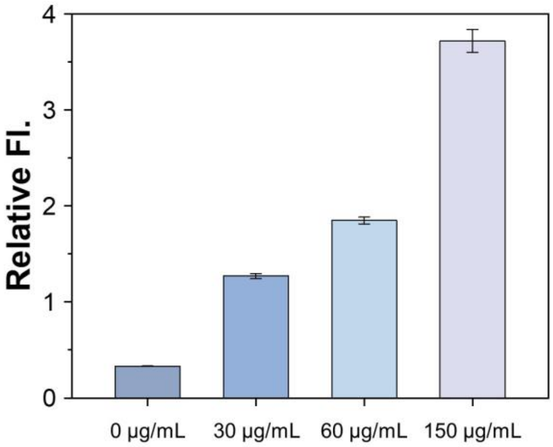

Figure S23. Relative fluorescence intensity of zebrafish cultured with different concentrations of HL-PS-COOH. Data are presented as mean ± SD (n = 3).

References

[1] A. Moraz, F. Breider, Detection and quantification of nonlabeled polystyrene nanoparticles using a fluorescent molecular rotor, Anal. Chem. 93 (2021) 14976-14984, https://doi.org/10.1021/acs.analchem.1c02055.

[2] B. Chaisrikhwun, M. J. D. Balani, S. Ekgasit, et al., A green approach to nanoplastic detection: SERS with untreated filter paper for polystyrene nanoplastics, Analyst 149 (2024) 4158-4167, https://doi.org/10.1039/D4AN00702F.

[3] S. Xiao, A. Filippini, M. Casadei, et al., Fast and portable fluorescence lifetime analysis for early warning detection of micro- and nanoplastics in water, Environ. Res. 244 (2024) 117936, https://doi.org/https://doi.org/10.1016/j.envres.2023.117936.

[4] Y. Lu, T. Ji, W. Xu, et al., Rapid, sensitive, and non-destructive on-site quantitative detection of nanoplastics in aquatic environments using laser-backscattered fiber-embedded optofluidic chip, J. Hazard. Mater. 479 (2024) 135591, https://doi.org/https://doi.org/10.1016/j.jhazmat.2024.135591.

[5] T. S. S. K. Naik, R. Varshney, P. C. Ramamurthy, Biomimetic iron-doped polydopamine sensor for selective detection of polystyrene nanoplastics, ACS. EST. Water 5 (2025) 3241-3250, https://doi.org/10.1021/acsestwater.5c00090.

[6] S. H. Lee, Y. Kim, Novel colorimetric detection of polypropylene nanoplastic via inhibition of salt-induced aggregation of gold nanoparticle, J. Ind. Eng. Chem. (2025), https://doi.org/https://doi.org/10.1016/j.jiec.2025.04.059.

[7] W. Guo, Y. Ma, D. Bai, et al., An electrochemiluminescence-activated amphiphilic perylene diimide probe: enabling highly sensitive and selective detection of polypropylene nanoplastics in the environment, Anal. Chem. 97 (2025) 10218-10226, https://doi.org/10.1021/acs.analchem.4c07054.

[8] Y. Liu, Y. Zhan, G.Wang, et al., Size-matching effects in quantitative detection of PS nanoplastics using controllable and reusable Ag nanoarrays SERS substrates, J. Hazard. Mater. 494 (2025) 138550, https://doi.org/https://doi.org/10.1016/j.jhazmat.2025.138550.
